# Effects of Cyberball on cognitive vulnerability for suicide in youth with a history of multiple suicide attempts

**DOI:** 10.3389/fpsyt.2026.1729109

**Published:** 2026-02-24

**Authors:** Myren N. Sohn, Signe L. Bray, Iliana Ortega, Alexander McGirr

**Affiliations:** 1 Department of Psychiatry, University of Calgary, Calgary, AB, Canada; 2 Mathison Center for Mental Health Research and Education, University of Calgary, Calgary, AB, Canada; 3 Hotchkiss Brain Institute, University of Calgary, Calgary, AB, Canada; 4 Alberta Children’s Hospital Research Institute, Alberta Children’s Hospital, Calgary, AB, Canada; 5 Department of Radiology, University of Calgary, Calgary, AB, Canada

**Keywords:** Cyberball, death/suicide implicit association test, decision making, emerging adults, social exclusion, suicide attempters

## Abstract

**Introduction:**

While the precipitants for suicide are varied, interpersonal stressors are commonly identified. We hypothesized that interpersonal stressors increase suicide risk by exacerbating cognitive vulnerabilities in decision-making, cognitive control, and implicit associations between the self and death/suicide.

**Methods:**

Interpersonal stress was modeled using the Cyberball paradigm in forty youth (16-24y) with a history of multiple (≥2) suicide attempts. Participants were randomized to either a social exclusion or overinclusion condition. Changes in mood and cognition were assessed before and after Cyberball using visual analog scales, the Game of Dice Task, the Iowa Gambling Task, the Balloon Analog Risk Test, the Word Color Stroop Test, and the Death/Suicide Implicit Association Test.

**Results:**

Social exclusion and overinclusion did not significantly impact decision-making, cognitive control or implicit association of the self with death/suicide, though high inter-individual variability was observed. Group differences were observed in the change in anger (*t*(34) = 2.47, *p* = 0.02), loneliness (*t*(34) = 2.56, *p* = 0.015), sadness (*t*(34) = 2.56, *p* = 0.02), and depression (*t*(34) = 2.25, *p* = 0.03).

**Conclusions:**

As compared to social overinclusion, Cyberball-induced social exclusion did not significantly influence performance on cognitive tasks associated with suicide risk. Future research may consider within-subject designs comparing exclusion and inclusion paradigms, using alternative acute stress manipulations or powering studies to detect smaller effect sizes when studying interpersonal stress in youth at high-risk for suicide.

## Introduction

Suicide is a leading cause of death in youth (1). Although it is difficult to predict who will die by suicide, individuals with a history of multiple suicide attempts are at a high risk of future suicide attempts and death by suicide (2, 3). While many factors may precede suicide, interpersonal stressors are common precipitants of suicidal crises, particularly in youth (4–7). Research illustrates differences in the emotional, neural, and physiological responses to interpersonal stressors in individuals with a history of attempting suicide compared to both healthy and psychiatric controls (8–16). This suggests that acute interpersonal stress may trigger cognitive processes associated with suicidal behaviors in this high-risk population. However, the specific cognitive domains that are impacted by interpersonal stress have not been well characterized in youth with a history of multiple suicide attempts.

Impulsivity, cognitive control, and decision making have been extensively investigated as cognitive and behavioral risk factors associated with suicide. These factors can differentiate individuals who have previously attempted suicide from those who have not (17–21). They have also been associated with the severity of suicide attempts (22, 23). Biases in decision-making may underlie a cognitive distortion towards considering reasons for, rather than against, suicide and deliberating for a shorter amount of time (24) to increase the likelihood of selecting suicidal behavior over healthier alternatives. In addition to measures of cognitive control and decision-making, recent developments have seen other means of assaying cognitive distortions relating to suicide using implicit association to identify suicide risk (25). The Death/Suicide-Implicit Association Test (Death/Suicide-IAT) uses the Stroop effect to measure differences in the strength of implicit association of the self with life and death/suicide. Performance on this task has been associated with both past (25–33) and future suicidal behavior (25, 34, 35), as supported by meta-analysis (36). Although some data suggests that implicit associations with death/suicide are stable, other studies have shown that Death/Suicide-IAT performance differs with mental state [e.g., (37–39)]. We hypothesized that acute interpersonal stressors would exacerbate decision-making and other cognitive vulnerabilities to suicidal behaviors.

We sought to test this hypothesis using one of the most established laboratory-based social stressors: the Cyberball task. This task is a computerized ball tossing game where the researchers manipulate the degree to which a participant is included or excluded from the game (40–42). Though findings have been mixed, research supports that social exclusion compared to inclusion elicits different emotional, neural, and physiological responses in individuals with a history of attempting suicide (11, 12, 15, 16). In this study, we examined how social exclusion compared to inclusion engages the cognitive processes of decision-making, cognitive control, and implicit associations with death/suicide in youth with a history of multiple suicide attempts.

## Methods

This study was approved by the University of Calgary Conjoint Health Research Ethics Board (REB21-1915). All participants provided written informed consent.

### Participants

Forty participants (16–24 years) were recruited between April 2022 and August 2024 from outpatient clinics associated with the University of Calgary. The inclusion criteria were: a history of at least two previous suicide attempts as defined by the Columbia Suicide Severity Rating Scale [CSSRS; (43)], the ability to provide informed consent, and parent/guardian supervision for at least 24 hours after the experiment. Exclusion criteria consisted of previous exposure to the Cyberball task, the inability to complete computerized cognitive tests (e.g., motor deficits, color-blindness), current suicidal ideation with intent, or an actual suicide attempt within the past month. When applicable, participants were asked to complete a 48-hour washout of stimulants and other as-needed psychiatric medications that may influence cognitive task performance (e.g., benzodiazepines). Other daily psychiatric medications were continued as usual. Medical records were reviewed for psychiatric diagnoses. We did not control for time of last meal; however, participants completed both sessions around the same time of day (morning or afternoon).

### Measures

#### Clinical assessments

Clinical interviews consisted of the CSSRS (43) and the Karolinska Interpersonal Violence Scale [KIVS (44)]. The CSSRS was used to characterize our sample and confirm eligibility, including number and timing of past suicide attempts and the frequency and severity of lifetime suicidal ideation. The KIVS was used to identify participant perpetration and victimization of violence across childhood (6-14years) and adulthood (15 years and older) (44). The North American Adult Reading Test (NAART) was used to measure general intelligence. Using NAART scores, we present estimates of full-scale IQ based on the equation in Blair & Spreen, suggesting that calculated estimates are significantly associated with actual IQ [*r* = 0.75, *p* < 0.001; (45)]. All interviews were conducted by a trained PhD student (MNS).

#### Self-reports

The sample was characterized using self-reported questionnaires including a demographics form, the Adverse Childhood Experiences questionnaire [ACE; (46)], the Autism Quotient [AQ; (47)], the Quick Inventory of Depressive Symptoms-Self Report [QIDS-SR; (48)], the Borderline Symptom List-23 [BSL-23, (49)], the Difficulty in Emotional Regulation Scale [DERS; (50)], and the Barratt Impulsivity Scale-11; [BIS-11; (51)]. Suicidal ideation over the past 48-hours was assessed using the self-report version of the Beck Scale for Suicidal Ideation [BSI, (52)].

To assess the efficacy of the Cyberball manipulation, the Need Threat Scale [NTS; (42)] was used to measure self-reported need satisfaction immediately after Cyberball. Higher scores on the NTS indicate higher need satisfaction (lower need threat). Visual Analog Scales (VAS) were also used to measure self-reported mood “in this moment” with ratings from 0 (not feeling this emotion at all) to 100 (feeling this emotion the most you could ever imagine). VAS assessments included sadness, depression, anger, loneliness, suicidal ideation, anxiety, and hope for the future.

#### Cognitive testing

The battery of cognitive tests selected for this study focused on probabilistic decision-making given its repeated implication in suicide risk, implicit association with death/suicide, and control tasks to account for non-specific effects. All cognitive tasks were completed using Inquisit 5 software (https://www.millisecond.com/download) on a 13.5” Acer Swift3 Windows laptop.

*Effort: Finger Tapping Test (FTT; ~7-minutes)*. The FTT (53) is a measure of malingering and was completed as a measure of effort throughout testing sessions (53). Participants were excluded if they displayed low effort on the FTT defined as a dominant-hand score ≤28 in females or ≤35 in males (53). The FTT was completed during the baseline assessment (session 1), immediately before the Cyberball task (start of session 2) and at the end of the post-Cyberball assessment (end of session 2).

*Decision-Making: Game of Dice Task (GDT; ~6-minutes).* The GDT measures a tendency towards making risky versus safe decisions when given explicit and stable rules but uncertain outcomes (54). Suicide attempters make riskier decisions than non-attempters on this task (17) and performance may worsen following social exclusion as compared to inclusion in healthy individuals (55). Participants are told that their goal is to win as much money as possible when betting on the outcome of a die roll. They are given two “risky” options [low probability 1/6 or 2/6, high payoff: $1,000 or $500] and two “safe” options [high probability: 3/6 or 4/6, low payoff: $200 or $100]. The net score is the difference between the number of safe and risky selections. Scores range from 0 to 18, with lower scores being interpreted as riskier decision making. The GDT has a test-retest correlation of *r* = 0.49 over a three-week interval (56). To minimize the impact of practice effects on change scores, participants completed a double baseline during the first session (57). Only the second baseline was considered in analyses.

*Decision-Making: Iowa Gambling Task (IGT; ~3.5 minutes).* The IGT measures a tendency towards risky decision making when both the rules of the game and the outcomes are uncertain, thus, requiring explicit learning in conditions of uncertainty (58, 59). Participants are presented with four decks of cards and $2000. They are told that some decks may be more profitable than others and they have 100 chances to win as much money as possible. Two decks are advantageous (more winnings) while two are disadvantageous (more losses). The net score is the difference between the number of advantageous and disadvantageous selections. A lower score represents more disadvantageous choices. To minimize the impact of practice effects on change scores, participants completed two different versions of the IGT in a random, counter-balanced order. The versions were identical except for the placement of the advantageous and disadvantageous decks. Test-retest reliability of the IGT is *r* = 0.60 within a single session (60) and ranges from *r* = 0.35 to *r* = 0.65 over 2-weeks (61). Suicide attempters make riskier decisions than non-attempters on the IGT [Meta-analysis: (18)] and acute interpersonal stress may result in worse performance in healthy controls, though findings have been mixed (55).

*Decision-Making: Balloon Analog Risk Task (BART; ~8-minutes).* The BART measures impulsivity and aversion to risk (62). Participants are given 30 balloons and with each balloon, they have a choice to either continue pumping up the balloon, earning $0.05 per successful pump, or to collect their earnings. If a balloon pops before the earnings are collected, the money earned on that balloon is lost. The primary outcome is the average number of pumps on unexploded balloons (“adjusted average pump count”). Higher scores are interpreted as riskier behaviors. The BART has good test-retest reliability with a correlation *r* = 0.69 over a 3-week interval (56). Suicide attempters may make more impulsive decisions on the BART (63), though performance may be less sensitive to the effects of social exclusion in healthy controls (55). To minimize the impact of practice effects on change scores, participants completed a double baseline during the first session (57). Only the second baseline was considered in analyses.

*Cognitive Control: Stroop Word-Color Task (SWCT; ~3-minutes).* Cognitive control was assessed using the SWCT with keyboard responding (64). The SWCT asks participants to press a key indicating either the color of the font a word is written in or the meaning of the word and measures the speed and accuracy of each response. On congruent trials, the color of the text and the meaning of the word are the same while these differ on incongruent trials. The primary outcome is the interference score [Interference=Incongruent-Congruent reaction times]. The test-retest reliability of interference scores is *r* = 0.46 over a 1-week interval (65). Suicide attempters perform worse on the SWCT compared to non-attempters [meta-analysis: (18)] and acute interpersonal stress impairs cognitive control in healthy relatives of suicide decedents (66). To minimize the impact of practice effects on change scores, participants completed a double baseline during the first study session (57). Only the second baseline was considered for analyses.

*Implicit Associations: Death/Suicide-Implicit Association Test (Death/Suicide-IAT; ~5.5-minutes).* Implicit associations between the self and death/life were measured using the Death/Suicide-IAT (25). This task measures latency in sorting words to their target category (Life/Death) when each category is paired with an attribute dimension (Me/Not Me), creating an implicit association between the attribute dimension and the target category. The strength of association between concepts is measured using a composite standardized mean difference score (D-Score) for “Me-Life/Not Me-Death” and “Me-Death/Not Me-Life” (67). D-Scores range from -2 to +2 with zero indicating an equivalent strength of implicit associations with life and death. Positive scores favor “Me-Death” and “Not Me-Life” while negative scores favor “Me-Life” and “Not Me-Death”. Implicit association tests have an average test re-test reliability of *r* = 0.50 and internal consistency of α = 0.80 [meta-analysis: (68)]. Based on data suggesting relative stability of performance on the Death/Suicide-IAT (27, 69), we did not implement a double baseline procedure for this task.

### Cyberball task

Cyberball (42) is a computerized ball tossing game that consists of 30 throws in approximately 2-minutes. Participants were told that they were playing with two other participants, though in reality, the other players were computer generated. To increase realism, participants were allowed to select their favorite color to represent their computerized player and were told that other players were allowed to do the same. Though fair-play typically serves as a low-stress comparison to exclusion, individuals with borderline personality disorder tend to perceive fair-play similarly to social exclusion (70–73). Overinclusion, where participants receive 45% of ball tosses, has been shown to mitigate this effect (70). Due to the high prevalence of borderline traits in young suicide attempters (74, 75), we implemented an overinclusion paradigm where participants received 13/30 ball tosses. In the exclusion condition, participants received 3 ball tosses near the beginning of the game, before being completely excluded. Using a random number sequence, participants were randomized in a 1:1 ratio to experience social overinclusion (*n* = 20) or social exclusion (*n* = 20).

### Procedures

Participants attended two study visits on consecutive days. After providing informed consent, participants underwent a clinical interview to assess suicide histories (CSSRS) and histories of interpersonal violence (KIVS). During the first visit, participants completed a baseline cognitive testing battery and self-report questionnaires. To minimize practice effects, double baseline measures were implemented for the GDT, BART, and SWCT and two different versions of the IGT were completed in a counterbalanced order (57). Cognitive tasks were completed in a fixed order throughout both sessions: GDT, BART, SWCT, IGT, Death/Suicide-IAT. During the second session, we determined acute risk for suicide by measuring suicidal ideation within the last 48-hours. Participants then completed visual analog ratings of current mood and the FTT, before completing the Cyberball task (randomly assigned exclusion OR overinclusion). Immediately post-Cyberball, participants again completed mood ratings, cognitive tests, and additional self-report questionnaires. Participants were fully debriefed and provided with mental health resources at the end of the study.

### Statistical analyses

Independent samples t-tests and chi-squared tests were used to compare demographics and baseline characteristics between the overinclusion and exclusion groups. We planned to conduct independent samples t-tests to compare group differences in need satisfaction on the NTS immediately after Cyberball. However, if baseline characteristics differed between groups, a between-subjects analysis of covariance (ANCOVA) was conducted including these as covariates. Linear regressions were conducted to determine how group (overinclusion, exclusion) influenced change in mood (post-pre) on VASs. If baseline characteristics differed between groups, these were included as covariates in multiple linear regression analyses. Repeated measures (pre, post), between-subjects (overinclusion, exclusion) analyses of variance (ANOVAs) were conducted to compare group differences in the change in performance on measures of effort, decision-making, cognitive control, and implicit association tasks. If baseline characteristics differed between groups, repeated measures ANCOVAs were conducted including these as covariates. We conducted Benjamini-Hochberg False Discovery Rate (FDR) corrections for multiple comparisons.

## Results

### Sample characteristics

Forty youth (16-24y, M = 17.90, SD = 2.43) participated in this study. Participant sociodemographic and clinical characteristics are detailed in **Table 1**. The number of lifetime suicide attempts ranged from 2–20 with a median of three in each group (Z = 0.07, *p* = 0.94). Past 48-hour suicidal ideation was endorsed by *n* = 26 participants (BSI>0; overinclusion: *n* = 13, exclusion: *n* = 13). At baseline, individuals who endorsed past 48-hour suicidal ideation (BSI>0) had stronger implicit associations of the self with death/suicide (*M* = -0.15, *SD* = 0.28) compared to those without suicidal ideation (*M* = -0.43, *SD* = 0.23; *t (*38)=-3.14, *p* = 0.002, *mean difference* = -0.28, 95%CI: -0.45, -0.098). Baseline performance on other cognitive tasks did not significantly differ by endorsement of suicidal ideation (IGT: *mean difference* = 2.93, 95%CI: -15.94, 21.80; BART: *mean difference* = -6.49, 95%CI: -18.05, 5.06; GDT: *mean difference* = 5.06, 95%CI: -3.63, 13.74; SWCT: *mean difference* = 22.19, 95%CI: -83.84, 128.23). Considered continuously, BSI scores were skewed towards zero and distributions were similar in the overinclusion (Median = 5.00 [Range: 0-21]) and exclusion (Median = 5.50 [Range: 0-12]) groups (*Z* = 0.67, *p* = 0.51). Participants in both groups self-reported moderate depressive symptoms within the past week (*t*(38)=-1.19, *p* = 0.24).

**Table 1:** Participant characteristics.

Variables	Overinclusion n=20	Exclusion n=20	Statistics
*Age*	16.50y (16-24)	17.00y (16-22)	*Z =* -1.36, *p =* 0.18
*Cis Gender*	*n* = 12	*n* = 15	*X^2^* = 1.03, *p* = 0.31
*Female Sex*	*n* = 17	*n* = 17	
*Years of Education*	12.00 (10-16)	11.00 (10-18)	*Z =* 0.12, *p* = 0.91
*Caucasian*	*n* = 16	*n* = 13	*X^2^* = 1.13, *p* = 0.29
*Right-Handed*	*n* = 17	*n* = 18	*X^2^* = 0.23, *p* = 0.63
*English First Language*	85%	100%	*X^2^* = 3.24, *p* = 0.07
*NAART FSIQ*	107.60 (6.08)	106.87 (9.32)	*t*(36) = -0.29, *p* = 0.78
*Number Lifetime SA*	3.00 (2-20)	3.00 (2-18)	*Z* = 0.07, *p = 0.94*
*KIVS*	7.25 (4.08)	6.80 (3.37)	*t*(38) = -0.38, *p* = 0.71
*ACE*	4.00 (0-9)	4.00 (0-9)	*Z* = -0.27, *p* = 0.79
*BSI (past 48h)*	5.00 (0-21)	5.50 (0-12)	*Z* = 0.67, *p* = 0.51
*QIDS-SR*	14.40 (4.98)	12.75 (3.74)	*t*(38) = -1.19, *p* = 0.24
*BSL-23*	1.98 (0.91)	1.42 (0.67)	*t*(38) = -2.22, *p* = 0.03*
*BSL Behavioral Supplement*	3.50 (0-17)	2.00 (0-6)	*Z* = 0.93, *p* = 0.35
*Past Week Personal State*	49.69 (21.66)	51.00 (18.61)	*t*(38) = 0.21, *p* = 0.84
*AQ*	28.45 (5.37)	25.63 (5.32)	*t*(37) = -1.65, *p* = 0.11
*BIS-11*	77.60 (12.68)	68.20 (11.64)	*t*(38) = -2.44, *p* = 0.02*
*DERS*	120.10 (26.23)	99.50 (20.05)	*t*(38) = -2.79, *p* = 0.008*
*Taking Psychiatric Medications*	*n* = 16/20	*n* = 17/20	*X^2^* = 0.17, *p* = 0.68
*SSRI*	*n* = 10	*n* = 10	
*Stimulant*	*n* = 10	*n* = 7	
*Antipsychotic*	*n* = 7	*n* = 6	
*SARI*	*n* = 2	*n* = 3	
*SNRI*	*n* = 2	*n* = 2	
*Anticonvulsant*	*n* = 0	*n* = 4	
*Benzodiazepine*	*n* = 1	*n* = 2	
*NDRI*	*n* = 0	*n* = 2	
*Non-Benzodiazepine Hypnotic*	*n* = 0	*n* = 2	
*TCA*	*n* = 1	*n* = 0	
*Anti-adrenergic agent*	*n* = 0	*n* = 1	
*Psychiatric Diagnoses*	*n* = 17/19	*n* = 17/18	
*Major Depressive Disorder*	*n* = 7	*n* = 10	
*Persistent Depressive Disorder*	*n* = 3	*n* = 3	
*Bipolar Disorder*	*n* = 1	*n* = 2	
*ADHD*	*n* = 10	*n* = 9	
*Borderline Personality Disorder/Traits*	*n* = 6	*n* = 8	
*Generalized Anxiety Disorder*	*n* = 13	*n* = 13	
*Social Anxiety Disorder*	*n* = 4	*n* = 2	
*Adjustment Disorder*	*n* = 4	*n* = 3	
*Substance Use Disorder*	*n* = 0	*n* = 1	
*Conduct Disorder*	*n* = 0	*n* = 1	
*Oppositional Defiant Disorder*	*n* = 0	*n* = 2	
*Anorexia Nervosa*	*n* = 4	*n* = 0	
*Autism Spectrum Disorder*	*n* = 1	*n* = 2	
*Gender Dysphoria*	*n* = 3	*n* = 1	
*Unspecified-TSRD*	*n* = 5	*n* = 3	
*PTSD*	*n* = 2	*n* = 1	
*Learning Disability*	*n* = 1	*n* = 2	
*Intellectual Disability*	*n* = 0	*n* = 2	
*Obsessive-Compulsive Personality Disorder*	*n* = 1	*n* = 0	
*Obsessive Compulsive Disorder*	*n* = 1	*n* = 1	

Information on psychiatric diagnoses is based on *n* = 37, three participants did not provide consent to access medical records. Means (standard deviations) are provided for parametric data. Median (range) are provided for non-parametric data. Cisgender was defined as participants’ whose gender matched their sex assigned at birth. Past week personal state was measured on a visual analogue scale as part of the BSL-23: “Now we would like to know the quality of your overall personal state in the course of the last week. 0% means absolutely down, 100% means excellent. Please check the percentage which comes closest.”

NAART, North American Adult Reading Test; SA, Suicide Attempt; KIVS, Karolinska Interpersonal Violence Scale; ACE, Adverse Childhood Experiences; BSI, Beck Scale for Suicidal Ideation – Self Report. QIDS-SR, Quick Inventory of Depressive Symptoms-Self Report. BSL-23, Borderline Symptom List-23; AQ, Autism Quotient. BIS-11, Barratt Impulsivity Scale-11; DERS, Difficulties in Emotional Regulation Scale; ADHD, Attention-Deficit Hyperactivity Disorder; TSRD, Trauma and Stress Related Disorder; PTSD, Post-Traumatic Stress Disorder; SSRI, Selective Serotonin Reuptake Inhibitor; SNRI, Serotonin and Norepinephrine Reuptake Inhibitor; SARI, Serotonin Receptor Antagonist and Reuptake Inhibitor; NDRI, Norepinephrine and Dopamine Reuptake Inhibitor; TCA, Tricyclic Antidepressant.

Thirty-three participants reported taking any psychiatric medications at the time of assessment [*n = *17 reported taking stimulant medication (overinclusion: *n* = 10, exclusion: *n* = 7), all of whom withheld these medications on testing days]. No participants were excluded based on effort measured on the FTT. There were no significant changes in performance on the FTT in either group throughout study visits.

Symptoms of poor self-regulation and emotional lability significantly differed between groups. Participants in the overinclusion condition reported more past-week borderline personality symptoms (BSL-23: *t*(38)=-2.22, *p* = 0.03), higher levels of trait impulsivity (BIS-11: *t*(38)=-2.44, *p* = 0.02), and more difficulty with emotional regulation (DERS: *t*(38)=-2.79, *p* = 0.008). Accordingly, baseline scores for these three scales were included as covariates in all subsequent analyses.

### Effects of social exclusion and overinclusion on need satisfaction

A between subjects ANCOVA was conducted to determine how Cyberball group (overinclusion, exclusion) influenced self-reported need satisfaction on the NTS while controlling for group differences on the BSL-23, DERS, and the BIS-11. Controlling for these covariates, participants in the social exclusion (*M* = 2.01, *SD* = 0.61; *Marginal Mean* = 1.88, 95%CI: 1.59, 2.18) group reported significantly less need satisfaction than those in the overinclusion (*M* = 3.85, *SD* = 0.72; *Marginal Mean* = 3.98, 95%CI: 3.69, 4.28) condition (*F*(1, 35)=94.12, *p* < 0.001, *partial eta^2^* = 0.73; *mean difference* = -2.10, *p* < 0.001, 95%CI: -2.54, -1.66).

### Effects of social exclusion and overinclusion on self-reported mood

**Figure 1:**
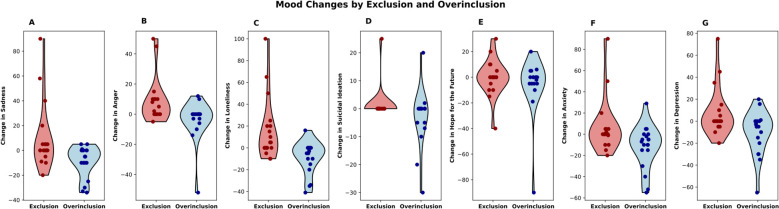
Self-reported changes in mood following social exclusion or overinclusion (change = post cyberball-baseline). Mood was measured on visual analog scales inquiring about **(A)** sadness, **(B)** anger, **(C)** loneliness, **(D)** suicidal ideation, **(E)** hope for the future, **(F)** anxiety, and **(G)** depression “in this moment”.

One participant in the overinclusion group did not complete baseline mood assessments resulting in a sample of *n* = 39 for these analyses. Separate multiple linear regressions were conducted to determine how Cyberball group assignment (overinclusion, exclusion) influenced change in mood immediately after Cyberball. While controlling for DERS, BSL-23, and BIS-11, the change in sadness (*t*(34)=2.56, *p* = 0.02), anger (*t*(34)=2.47, *p* = 0.02), loneliness (*t*(34)=2.561, *p* = 0.015), and depression (*t*(34)=2.25, *p* = 0.03) significantly differed between groups (**Figure 1**; **Table 2**). Changes in sadness, anger, and loneliness, but not change in depression, survived FDR correction. Changes in suicidal ideation (*t*(34)=1.93, *p* = 0.06), anxiety (*t*(34)=1.46, *p* = 0.15) and hope for the future (*t*(34)=1.25, *p* = 0.22) did not significantly differ between groups (**Figure 1**).

**Table 2:** Effects of social exclusion compared to overinclusion on mood and cognitive task performance.

Mood	Overinclusion mean rank	Exclusion mean rank	B	SE	t	p
Sadness	15.08	24.68	18.85	7.37	2.56	0.02*
Anger	14.97	24.78	12.74	5.15	2.47	0.02*
Loneliness	14.00	25.70	20.12	7.86	2.56	0.015*
Depression	15.76	24.03	17.06	7.59	2.25	0.03*
Suicidal Ideation	17.50	22.38	5.47	2.84	1.93	0.06
Anxiety	15.21	24.55	11.45	7.85	1.46	0.15
Hope for the future	18.84	21.10	8.40	6.75	1.25	0.22
*Cognition*	*Overinclusion* *M (SD)*	*Exclusion* *M (SD)*		*F*	*partial eta^2^*	*p*
IGT						
BL	-2.00 (27.10)	-10.10 (28.58)	Time	0.51		0.48
Post	-1.10 (37.89)	0.30 (41.79)	Time*Group	0.04	0.001	0.84
GDT						
BL	7.80 (10.95)	4.20 (14.84)	Time	0.002		0.97
Post	7.10 (10.39)	8.00 (12.77)	Time*Group	2.21	0.06	0.15
BART						
BL	35.50 (16.70)	30.76 (17.96)	Time	0.19		0.67
Post	38.31 (15.60)	34.43 (17.89)	Time*Group	0.39	0.01	0.54
SWCT						
BL	202.22 (159.78)	233.32 (155.35)	Time	0.12		0.73
Post	156.65 (122.32)	162.73 (158.12)	Time*Group	0.30	0.01	0.59
Death/Suicide-IAT						
BL	-0.26 (0.29)	-0.24 (0.30)	Time	0.21		0.64
Post	-0.27 (0.36)	-0.33 (0.28)	Time*Group	0.83	0.02	0.37

*p < 0.05. IGT, Iowa Gambling Task; GDT, Game of Dice Task; BART, Balloon Analog Risk Task; SWCT, Stroop Word Color Task; BL, baseline; Post, Post Cyberball; M, Mean; SD, Standard Deviation. Mood data: mean ranks of change scores.

### Performance on decision-making and cognitive control tasks is not influenced by social exclusion or overinclusion

**Figure 2:**
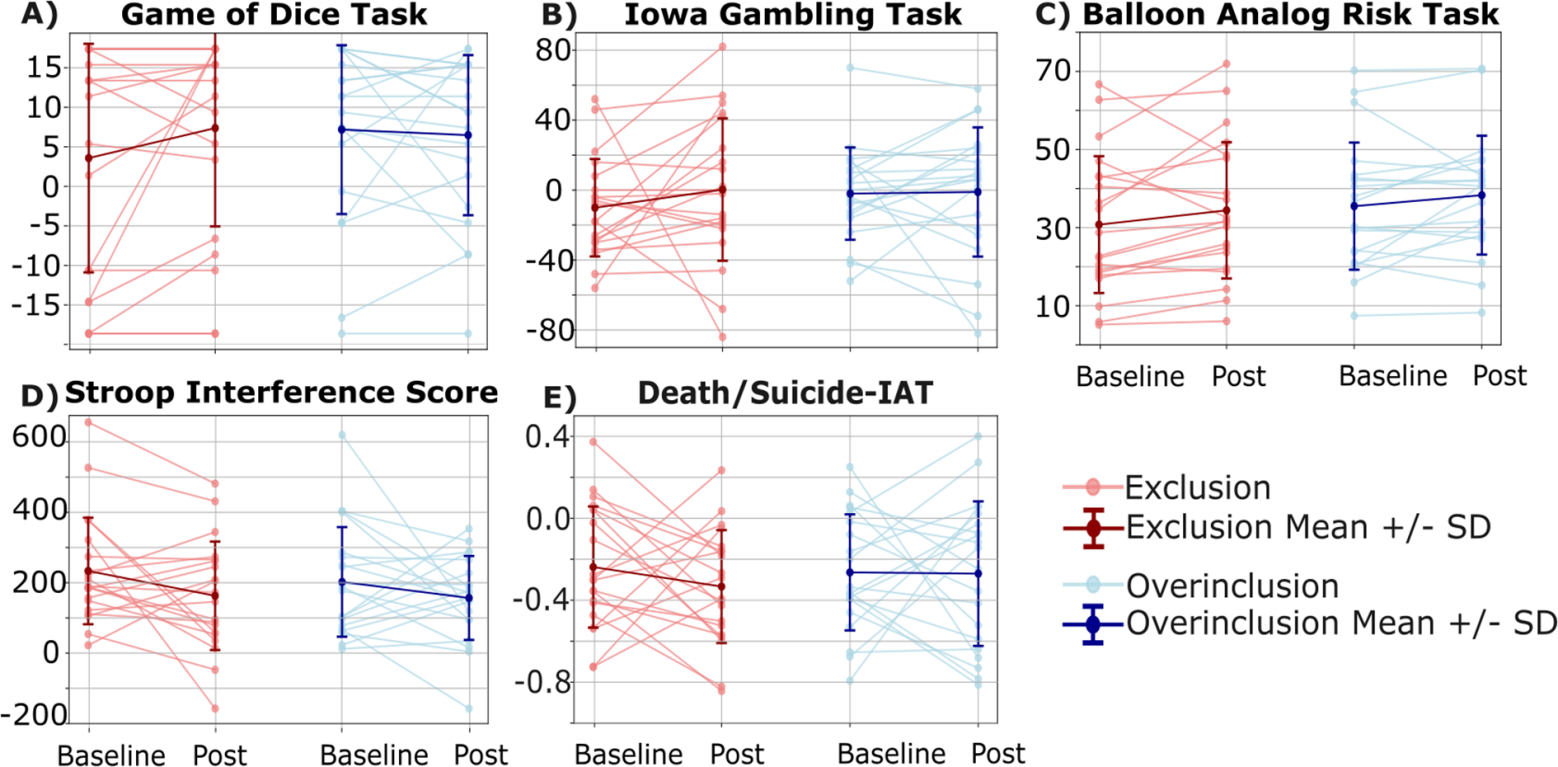
Cognitive task performance at baseline and post-Cyberball following social exclusion or overinclusion. Tasks examined decision-making: **(A)** Game of Dice Task, **(B)** Iowa Gambling Task, and **(C)** Balloon Analog Risk Task (BART); cognitive control: **(D)** Stroop Word-Color Task; and implicit associations with death/suicide: **(E)** Death/Suicide-Implicit Association Test (Death/Suicide-IAT).

Separate repeated-measures (TIME: pre, post) between-subjects (GROUP: overinclusion, exclusion) ANCOVAs using BIS-11, BSL-23, and DERS scores as covariates, were conducted to compare change in performance on the IGT, GDT, BART, and SWCT (**Figures 2A-D**; **Table 2**). Analyses revealed no main effect of time (*F*(1,35)=0.51, *p* = 0.48) nor a time*group interaction (*F*(1,35)=0.04, *p* = 0.84, *partial eta^2^* = 0.001) on IGT net scores. Net GDT performance did not differ over time (*F*(1,35)=0.002, *p* = 0.97) and there was no significant time*group interaction (*F*(1,35)= 2.21, *p* = 0.15, *partial eta^2^* = 0.06). The average adjusted pump count on the BART did not differ over time (*F*(1,35)=0.19, *p* = 0.67) and there was no significant time*group interaction (*F*(1,35)=0.39, *p* = 0.54, *partial eta^2^* = 0.01). SWCT interference scores did not differ over time (*F*(1,35)=0.12, *p* = 0.73) and there was no significant time*group interaction (*F*(1,35)=0.30, *p* = 0.59, *partial eta^2^* = 0.01).

### Performance on the death/suicide-IAT is not influenced by social exclusion or overinclusion

A repeated measures (TIME: pre, post) between-subjects (GROUP: overinclusion, exclusion) ANCOVA using BIS-11, BSL-23, and DERS as covariates was conducted to compare change in Death/Suicide-IAT performance between groups (**Figure 2E**). Analyses revealed no main effect of time (*F*(1,35)=0.21, *p* = 0.64) and no significant time*group interaction (*F*(1,35)=0.83, *p* = 0.37, *partial eta^2^* = 0.02).

## Discussion

In this study, we used a controlled laboratory stressor to test how implicit and cognitive risk factors for suicide change following acute stress among youth at high risk for suicide. Using the Cyberball paradigm, youth were either excluded or over-included. Neither of these conditions impacted decision-making, cognitive control, or implicit associations with death/suicide. Interestingly, the effects of social exclusion and overinclusion on mood were small and driven by decreases in negative affect in the overinclusion group. While no systematic changes were observed on cognitive tasks and changes in self-reported mood were small, the social exclusion group reported significantly less need satisfaction compared to the overinclusion group immediately after Cyberball, suggesting that the manipulation had the intended effect.

Our negative findings should be interpreted considering our statistical power. The current study was powered to detect a moderate effect size based on studies in non-suicidal participants where social exclusion has been shown to impact decision-making [i.e., (55, 76)]. Two assumptions with respect to the generalizability of effects observed in non-suicidal individuals to those with a history of multiple suicide attempts deserve highlighting. First, by selecting individuals at very high-risk for suicide by virtue of multiple previous suicide attempts, the specific vulnerabilities may have reached a ceiling effect such that the effects of social exclusion are muted when baseline task performance already reflects riskier decision-making, poorer cognitive control, and stronger implicit associations with death/suicide than observed in healthy individuals (17, 77–79). This is consistent with our visual analog data suggesting that the largest effect observed in this population is from a reduction in negative affect in the social overinclusion condition (**Figure 1**). In a related assumption, we selected participants for a vulnerability to suicide based on a history of multiple prior suicide attempts, a common means of studying suicide risk (80). However, considering that most people who die by suicide do so on the first attempt (81), our population may have resiliency or alternative vulnerabilities to suicide that are not impacted by interpersonal stress. The effects of acute interpersonal stress are complex [e.g. (9, 10)], and few authors have stratified groups by a history of multiple suicide attempts. One study similarly reported a blunted effect of social exclusion on decision-making in multiple as compared to single suicide attempters, however, cognitive testing was not completed at baseline (12). The effects of acute stressors may be further complicated by characteristics like impulsivity (9, 82) and real-life experiences of interpersonal stress (83). Importantly, randomization in our study did not achieve comparable baseline characteristics, and although we statistically accounted for these differences, matched samples would be required to definitively rule out their influence.

Accordingly, individual differences in interpersonal stress-sensitivity may have impacted our results. The variability observed in both the changes in cognitive task performance and mood ratings emphasizes individual differences in the response to interpersonal stress. While interpersonal stress is an important proximal risk factor for suicide, it is not the only stressor that precipitates suicidal behaviors and different people are vulnerable to different types of stress (84, 85). Indeed, the magnitude of the stress response may differ based on factors such as current suicidal ideation, histories of violence and abuse, borderline symptoms, difficulties with emotional regulation, and impulsivity (8, 70, 86–89). As these characteristics differed between groups, they may have influenced participant susceptibility to the effects of their assigned intervention. Future studies might control for such differences by implementing within-subjects designs examining the full continuum of social inclusion and exclusion (41, 90). Moreover, Cyberball is a mild form of interpersonal stress which, in some, may not accurately model interpersonal stress in a way that is relevant to suicide risk. It is possible that other laboratory based interpersonal stressors would have had a different effect. However, across studies using different interpersonal stress paradigms, inter-individual variability remains common [e.g., (9)].

The features of the Cyberball task design should also be considered. While a review of 120 studies in healthy individuals suggests that versions of the Cyberball manipulations using 30-ball tosses (<5-minutes) are sufficient in eliciting effects on mood, feelings of exclusion, and threat to fundamental needs (40), the limited evidence supporting physiological and neural responses to social exclusion in suicide attempters comes from studies using longer versions of the paradigm. Specifically, 10-minutes of social exclusion resulted in a decrease in plasma oxytocin in undergraduate students with a history of suicide attempt (11). In euthymic adult females with a history of suicide attempt, 60-ball tosses per condition resulted in decreased blood-oxygen-level (BOLD) dependent signal in the left supramarginal gyrus and posterior insula during social exclusion compared to inclusion (15). Conversely, two studies using ≤30-ball tosses per condition did not show a mean difference between social exclusion and inclusion in reward preferences (12) or BOLD signal (14) in young adult and adolescent suicide attempters, respectively. Thus, a longer manipulation may be required to invoke more systematic effects of social exclusion in youth suicide attempters.

## Conclusions

There was no systematic effect of Cyberball-induced social exclusion nor overinclusion on measures of decision-making, cognitive control, or implicit associations with death/suicide in youth at high risk for suicide, however there was individual variation. Overall, the Cyberball literature would benefit from methodological standardization of the task (41, 90). Future studies in suicide attempters might consider recruiting larger samples, using within-subjects designs, or alternative acute stress paradigms to further examine the role of interpersonal stress in youth with a history of multiple suicide attempts.

## Data Availability

The raw data supporting the conclusions of this article will be made available by the authors, without undue reservation.
